# Blood Profile of Proteins and Steroid Hormones Predicts Weight Change after Weight Loss with Interactions of Dietary Protein Level and Glycemic Index

**DOI:** 10.1371/journal.pone.0016773

**Published:** 2011-02-14

**Authors:** Ping Wang, Claus Holst, Malene R. Andersen, Arne Astrup, Freek G. Bouwman, Sanne van Otterdijk, Will K. W. H. Wodzig, Marleen A. van Baak, Thomas M. Larsen, Susan A. Jebb, Anthony Kafatos, Andreas F. H. Pfeiffer, J. Alfredo Martinez, Teodora Handjieva-Darlenska, Marie Kunesova, Wim H. M. Saris, Edwin C. M. Mariman

**Affiliations:** 1 Department of Human Biology, NUTRIM School for Nutrition, Toxicology and Metabolism, Maastricht University Medical Centre, Maastricht, The Netherlands; 2 Institute of Preventive Medicine, Copenhagen University Hospital, Copenhagen, Denmark; 3 Department of Clinical Biochemistry, Copenhagen University Hospital, Gentofte, Denmark; 4 Department of Human Nutrition, Faculty of Life Sciences, University of Copenhagen, Copenhagen, Denmark; 5 Department of Clinical Chemistry, Maastricht University Medical Centre, Maastricht, The Netherlands; 6 Elsie Widdowson Laboratory, MRC Human Nutrition Research, Cambridge, United Kingdom; 7 Department of Social Medicine, Preventive Medicine & Nutrition Clinic, University of Crete, Crete, Greece; 8 Department of Clinical Nutrition, German Institute of Human Nutrition, Nuthetal, Germany; 9 Department of Endocrinology, Diabetes and Nutrition, Charité Universitaetsmedizin Berlin, Berlin, Germany; 10 Department of Physiology and Nutrition, University of Navarra, Pamplona, Spain; 11 Department of Nutrition, Dietetics and Metabolic Diseases, National Multiprofile Transport Hospital, Sofia, Bulgaria; 12 Obesity Management Centre, Institute of Endocrinology, Prague, Czech Republic; University of South Florida College of Medicine, United States of America

## Abstract

**Background:**

Weight regain after weight loss is common. In the Diogenes dietary intervention study, high protein and low glycemic index (GI) diet improved weight maintenance.

**Objective:**

To identify blood predictors for weight change after weight loss following the dietary intervention within the Diogenes study.

**Design:**

Blood samples were collected at baseline and after 8-week low caloric diet-induced weight loss from 48 women who continued to lose weight and 48 women who regained weight during subsequent 6-month dietary intervention period with 4 diets varying in protein and GI levels. Thirty-one proteins and 3 steroid hormones were measured.

**Results:**

Angiotensin I converting enzyme (ACE) was the most important predictor. Its greater reduction during the 8-week weight loss was related to continued weight loss during the subsequent 6 months, identified by both Logistic Regression and Random Forests analyses. The prediction power of ACE was influenced by immunoproteins, particularly fibrinogen. Leptin, luteinizing hormone and some immunoproteins showed interactions with dietary protein level, while interleukin 8 showed interaction with GI level on the prediction of weight maintenance. A predictor panel of 15 variables enabled an optimal classification by Random Forests with an error rate of 24±1%. A logistic regression model with independent variables from 9 blood analytes had a prediction accuracy of 92%.

**Conclusions:**

A selected panel of blood proteins/steroids can predict the weight change after weight loss. ACE may play an important role in weight maintenance. The interactions of blood factors with dietary components are important for personalized dietary advice after weight loss.

**Registration:**

ClinicalTrials.gov NCT00390637

## Introduction

The worldwide epidemic of obesity and related health problems like diabetes [1] and others demands effective measures to help overweight and obese people to reduce their weight. However, to maintain a reduced weight is a challenge because the majority of people regain weight in the long term [2]. Targeting the obesity problem by dietary intervention, a pan-European project ‘Diogenes’ studied the relative efficacy of four different diets with variation in protein/carbohydrate content and glycemic index (GI) with respect to weight loss maintenance [3], [4]. It showed that both a modestly higher protein content and a modest reduction in GI improve weight loss maintenance [5].

It is well recognised that obesity has both a genetic and an environmental basis. An individual's susceptibility is determined in part by genetics, while the observed outcome is strongly influenced by environmental factors (diet, physical activity etc.). In addition to single-gene variants causing obesity [6], there is growing evidence that gene-environment interactions influence the development of obesity and the weight change by interventions [7], [8], [9]. An accurate assessment of genetic background comes from genotyping and transcription measurements. However, blood proteins offer an indirect assessment, since their circulating levels are determined by genetic and environmental factors.

The development of obesity is a complex physiological process, so are weight loss and weight maintenance. Previous studies on the prediction of weight change after weight loss have mostly focussed on psychological and behavioural aspects [10], [11], with some studies addressing biological aspects [8], [12]. Profiling of blood proteins covering various functions may allow us to predict the weight maintenance more accurately. Here we investigated the blood profile of women who participated in the Diogenes dietary intervention study with 31 proteins and 3 steroid hormones from various functions that have been shown to be related to obesity. The profiling of these targeted adipokines, cytokines, inflammation markers, vascular factors, satiety hormones, sex hormones and other metabolic hormones, allowed us to evaluate the prediction power of these blood analytes for weight change after weight loss, and with respect to possible interaction with dietary protein and GI levels.

## Methods

### Participants and study design

The participants were part of the pan-European, randomized and controlled dietary intervention study Diogenes (http://www.diogenes-eu.org). The details on design and dietary intervention were reported previously [3], [4]. In brief, from clinical investigation day (CID) 1, overweight or obese but otherwise healthy subjects followed an 8-week low calorie diet (LCD) with about 3.3 MJ/d, and participated in clinical investigation on CID2 at the end of this weight loss period. Those who achieved ≥8% loss of initial body weight were in a 2×2 factorial design randomized to one of the following four moderate-fat diets or a control diet to be consumed ad libitum for 6-month weight maintenance with dietary counselling every 2–4 weeks [3], [4]: low protein (LP) and low GI (LGI) (LP/LGI), LP and high GI (HGI) (LP/HGI), high protein (HP) and LGI (HP/LGI), HP and HGI (HP/HGI). Participants were advised to maintain body weight, but there were no restrictions with respect to further weight loss. At the end of this weight maintenance period the participants underwent a further clinical investigation day (CID3).

On each CID, the anthropometrical and physiological parameters were measured, and blood, urine and fat biopsies were taken using the same standardized protocol at each centre [4]. For the present research, EDTA plasma and serum samples were obtained from overnight-fasting participants. The samples were aliquoted and kept at −80°C during storage and transportation. In addition, serum glucose, triglycerides, cholesterols, dietary intake based on food diary, and urinary analysis on 24-hr nitrogen excretion to assess adherence to the diet were measured as previously described [3], [4], [5].

The sample size estimation was done based on the complete Diogenes study and has been described previously [5]. For reason of power, we focused on female participants in each of the four maintenance diet groups. The dietary interventions were completed by 236 adult Caucasian women who were below 50 years of age, non-diabetic and non-dyslipidemic with fasting glucose <7mM, triglyceride <3.6mM and total cholesterol <7mM at CID1. A ‘weight maintenance score’ of relative weight change over the initial weight loss was calculated to assess the outcome of weight maintenance. In this way the influence of weight loss on weight maintenance is taken into account.
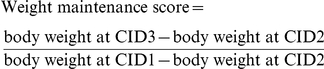



The subjects beyond the 10–90 percentiles of the score of each diet group were considered as the extremes and excluded from this analysis. From the remaining subjects, the 12 with the lowest (negative) score were defined as the weight-losers and the 12 with highest (positive) score as the weight-regainers in each diet group. In total, 96 subjects were selected for our study.

### Measurement of targeted blood factors

We first composed a large list of blood proteins involved in obesity as reported in the literature, then searched for available assay methods. The targeted analytes were mainly determined by its relevance for obesity as described in **Text S1**.

All the samples were blinded and randomly allocated with respect to dietary intervention and weight change prior to transport to the labs for analysis. The majority of the candidates were analyzed in plasma, unless otherwise stated in serum, by two multiplex biomarker testing laboratories with Clinical Laboratory Improvement Amendments (CLIA) certification: Rules Based Medicine (RBM; Austin, TX, USA) applying their Human Metabolic Map version 1.0, (http://www.rulesbasedmedicine.com/products-services/human-metabolic.asp), and SearchLight (Aushon BioSystems, Woburn, MA, USA) applying a customized multiplex immunoassay.

Interleukin (IL) 6, IL8 and tumor necrosis factor (TNF) α were analyzed with Sanquin Pelikine compact human ELISA kits (Amsterdam, the Netherlands). Amylin (IAPP) was analyzed with Linco human amylin (total) ELISA kit (St. Charles, MO, USA). Matrix metalloproteinase 9 (MMP9) was analyzed in serum by R&D systems Quantikine human MMP-9 (total) immunoassay kit (Minneapolis, MN, USA). Serum haptoglobin (HPT) was determined by a clinical immunoturbidimetric method using an LX-20 analyzer (Beckman-Coulter, Brea, CA, USA). Serum C-reactive protein (CRP) was quantified by an immunoturbidimetric assay with monocloncal antibodies (Roche Diagnostics, Hvidovre, Denmark) using a COBAS Integra 400 analyzer. Fibrinogen (FG) and coagulation factor VII (F7) concentrations were determined only at CID1 by measuring the clotting time of the diluted plasma with the STA-R Evolution Coagulation Analyzer (Diagnostica Stago, Asnieres Sur Seine, France).

### Ethics

The study was approved by local ethical committees in the respective countries: 1. Medical Ethics Committee of the University Hospital Maastricht and Maastricht University, the Netherlands; 2. The Committees on Biomedical Research Ethics for the Capital region of Denmark, Denmark; 3. Suffolk Local Research Ethics Committee, United Kingdom; 4. University of Crete Ethics Committee, Greece; 5. the Ethics Commission of the University of Potsdam; 6. Research Ethics Committee at the University of Navarra, Spain; 7. Ethical Committee of the Institute of Endocrinology, Czech Republic; 8. Ethical Committee to the National Transport Multiprofile Hospital in Sofia, Bulgaria. All participants signed a written informed consent.

### Data analysis

Analytes by multiplex assays were excluded if more than half of the samples were not measurable on the standard curve or if controls showed high variation. The final list of included analytes is shown in **Table** 1. The values of insulin-like growth factor (IGF) 1 of 16% of the samples and the values of growth hormone (GH) of 6% of the samples, which were flagged as being below the detection limit, were imputed with a value of half of the lowest detected concentration. The data of plasma insulin (INS) measured by RBM were calibrated using insulin data from a subset of serum samples measured by a solid-phase, two-site chemiluminescent immunometric assay (Siemens Medical Solutions Diagnostics, Ballerup, Denmark) using an Immulite 2500 analyzer. Further, outliers defined as a data point out of the mean±4SD range were removed per analyte.

**Table 1:** List of analyzed blood proteins and steroid hormones.

Category	Symbol	Name	Executed
Sex hormones	PRO	Progesterone	Rules Based Medicine
	TES	Testosterone	Rules Based Medicine
	LH	Luteinizing Hormone	Rules Based Medicine
	FSH	Follicle Stimulation Hormone	Rules Based Medicine
	PRL	Prolactin	Rules Based Medicine
Other steroid hormone	COR	Cortisol	Rules Based Medicine
Vascular factors	ACE	Angiotensin I converting enzyme 1	Rules Based Medicine
	AGT	Angiotensinogen	Rules Based Medicine
	PAI1	Plasminogen activator inhibitor-1, active	Aushon SearchLight
	FG	Fibrinogen	In-house
	F7	Coagulation factor VII	In-house
Adipokines	LEP	Leptin	Rules Based Medicine
	RETN	Resistin	Rules Based Medicine
	ASP	Acylation stimulation protein	Rules Based Medicine
	ADIPOQ	Adiponectin	Rules Based Medicine
	RBP4	Retinol binding protein 4	Aushon SearchLight
Insulin and related hormones	INS	Insulin	Rules Based Medicine
	GCG	Glucagon	Rules Based Medicine
	IAPP	Islet amyloid polypeptide, amylin, total	In-house
Immunoproteins	MIF	Macrophage migration inhibiting factor	Aushon SearchLight
	IL6	Interleukin 6	In-house
	IL8	Interleukin 8	In-house
	TNFα	Tumor necrosis factor alpha	In-house
	MMP9	Matrix metallopeptidase 9	In-house^1^
	HPT	Haptoglobin	In-house^1^
	CRP	C-reactive protein	In-house^1^
Growth factors	GH	Growth hormone	Aushon SearchLight
	IGF1	Insulin-like growth factor 1	Rules Based Medicine
	VEGFD	Vascular endothelial growth factor-D	Aushon SearchLight
	PEDF	Pigment epithelium-derived factor	Aushon SearchLight
	IGFBP1	Insulin-like growth factor binding protein 1	Aushon SearchLight
	IGFBP3	Insulin-like growth factor binding protein 3	Aushon SearchLight
Satiety hormones	GLP1	Glucagon-like Peptide-1, total	Rules Based Medicine
	PP	Pancreatic polypeptide	Rules Based Medicine

1 Analyzed in serum, others in plasma.

The anthropometrical and physiological parameters were expressed as mean±SD. Student t-test or Mann-Whitney test was applied to compare the difference between weight-losers and -regainers.

Taking weight loss or regain during the 6-month maintenance period as the outcome, logistic regression with Logit function in Generalized Linear Model was used to examine blood analytes one by one with the concentrations at CID1, CID2, and the fold change during the weight loss period (CID2/CID1) (all Ln-transformed), with age and the fold change of weight as covariates. When the interactions of blood analytes with dietary components were examined, the dietary protein level and GI level were also included as factors in the regression model. Significant variables were further used to build multinomial logistic regression models by backward stepwise modelling, with age and the fold change of weight always as forced entry variables. Nagelkerke pseudo R^2^ was used to estimate the explained variance by the prediction model. The above analyses were done with SPSS version 15.0 (SPSS Inc., Chicago, IL, USA). A two-sided p-value<0.05 was taken as significant.

Random Forests (RF) is a supervised non-linear and non-parametric learning algorithm, which has been successfully applied to various, especially biological problems and with a good reputation on accuracy and robustness [13]. In RF, out-of-bag (oob) error rate estimation warrants no need for cross-validation or a separate test set to get an unbiased estimation of the test set error. Mean Decreased Gini (MDG) and Mean Decreased Accuracy (MDA) are indices of the importance of the variable in the classification. This was done using ‘randomForest’ package version 4.5–34 [14] with R version 2.10.1 [15]. Prior to RF analysis, the missing values were imputed using the Probabilistic PCA (PPCA) method and all values were normalized by being centred to the mean and divided by the standard deviation of each variable with a web server from MetaboAnalyst [16].

## Results

### Subjects' characteristics

From the Diogenes dietary intervention study, 96 overweight/obese but otherwise healthy women (29–49 years of age) who had most pronounced (but not extreme) continued weight loss or weight regain after weight loss according to the weight maintenance score, were selected evenly from 4 dietary groups. Weight-losers lost 3.3±2.2 kg weight, while weight-regainers regained 3.9±1.2 kg weight during the 6-month maintenance period.

Anthropometrical and physiological characteristics were not different at baseline (CID1) and post-weight loss (CID2), and only showed a trend (p = 0.057) for younger age in the weight-losers compared to the -regainers (**Table** 2). During the weight loss period, the fold change of weight was the only one different, although borderline, between the weight-losers (0.89±0.03) and -regainers (0.90±0.02, p = 0.021). Therefore, age and the fold change of weight were always controlled in the following logistic regression analyses.

**Table 2:** Characteristics of study subjects.

Parameters	Time	Pooled		Diet1 LP/LGI		Diet2 LP/HGI		Diet3 HP/LGI		Diet4 HP/HGI	
		WLn = 48	WRn = 48	WLn = 12	WRn = 12	WLn = 12	WRn = 12	WLn = 12	WRn = 12	WLn = 12	WRn = 12
Weight maintenance score	**−0.28±0.14**	**0.42±0.12**	**−0.31±0.11**	**0.43±0.10**	**−0.17±0.10**	**0.52±0.11**	**−0.36±0.20**	**0.38±0.11**	**−0.30±0.08**	**0.33±0.10**	
Age (year)		39.3±5.3	41.3±5.0	43.0±5.8	41.1±3.8	**38.3±3.6**	**42.9±3.6**	38.4±4.4	39.3±6.0	37.4±5.6	41.8±6.0
Weight (kg)	CID1	97.5±16.4	93.3±12.6	101.3±18.5	98.9±10.8	87.3±6.9	91.9±12.8	104.5±16.4	91.6±16.2	96.7±17.3	91.0±9.3
	CID2	86.3±14.6	83.8±11.8	89.7±17.5	88.7±9.8	78.0±7.6	83.1±12.6	91.7±14.7	81.7±15.3	85.8±14.4	81.5±8.4
	CID3	83.0±13.6	87.7±12.2	86.1±16.9	93.2±10.4	**76.5±7.8**	**87.5±12.5**	87.1±13.5	85.4±15.9	82.4±13.6	84.7±8.2
BMI (kg·m^−2^)	CID1	34.5±4.8	33.3±4.3	35.8±5.0	34.6±4.4	30.9±2.6	33.4±4.4	**36.3±4.2**	**32.2±4.5**	35.0±5.4	33.0±4.2
	CID2	30.6±4.3	29.9±4.1	31.6±4.9	31.1±4.2	27.6±2.9	30.2±4.4	31.9±3.8	28.8±4.2	31.1±4.5	29.5±3.7
	CID3	**29.4±4.0**	**31.3±4.2**	30.4±4.8	32.6±4.3	**27.1±2.9**	**31.8±4.5**	30.3±3.3	30.0±4.3	29.8±4.2	30.6±3.7
Waist (cm)	CID1	104±13	102±11	107±13	107±10	96±7	101±13	107±9	101±13	105±19	99±10
	CID2	95±12	94±10	96±12	98±9	**86±7**	**94±9**	100±7	92±13	96±16	93±9
Systolic blood pressure (mmHg)	CID1	121±13	120±12	128±12	122±13	114±10	116±13	116±12	119±11	124±12	124±13
	CID2	116±12	114±13	123±12	115±12	110±11	110±15	113±10	118±11	119±14	114±13
Diastolic blood pressure (mmHg)	CID1	75±10	73±10	80±11	75±9	71±8	70±12	74±6	73±11	75±13	75±8
	CID2	71±9	71±11	73±7	74±12	69±11	68±8	70±8	75±12	73±11	69±10
Cholesterol (mmol/L)	CID1	4.6±0.8	4.6±1.0	5.0±0.8	4.3±1.4	4.6±0.8	4.9±0.9	4.5±0.9	4.6±0.7	4.5±0.7	4.7±1.0
	CID2	4.1±0.7	4.2±0.8	4.3±0.8	4.0±1.0	**3.9±0.4**	**4.4±0.6**	4.1±0.4	4.0±0.9	4.0±0.9	4.2±0.8
Triglycerides (mmol/L)	CID1	1.3±0.5	1.2±0.5	1.4±0.6	1.2±0.6	1.1±0.4	1.1±0.6	1.2±0.7	1.0±0.4	1.2±0.3	1.5±0.5
	CID2	1.1±0.3	1.1±0.5	1.1±0.4	1.2±0.8	1.0±0.4	1.0±0.4	1.1±0.3	0.9±0.3	1.1±0.3	1.3±0.4
HDL (mmol/L)	CID1	1.3±0.3	1.3±0.4	1.3±0.3	1.1±0.4	1.3±0.3	1.4±0.3	1.3±0.3	1.4±0.4	1.2±0.2	1.1±0.3
	CID2	1.2±0.2	1.2±0.3	1.2±0.3	1.1±0.3	1.2±0.3	1.3±0.2	1.2±0.2	1.3±0.4	1.1±0.2	1.1±0.2
LDL (mmol/L)	CID1	2.8±0.7	2.8±0.8	3.1±0.7	2.7±1.1	2.7±0.7	3.0±0.7	2.6±0.7	2.7±0.6	2.8±0.6	2.9±0.9
	CID2	2.4±0.6	2.5±0.7	2.6±0.8	2.4±0.7	2.3±0.4	2.7±0.6	2.4±0.3	2.3±0.7	2.4±0.8	2.6±0.8
Glucose (mmol/L)	CID1	4.9±0.8	5.0±0.6	5.2±0.7	5.4±0.9	4.7±0.4	4.8±0.4	4.8±0.5	5.0±0.5	4.8±1.2	5.0±0.6
	CID2	4.7±0.7	4.8±0.4	4.9±1.0	4.9±0.6	4.5±0.4	4.7±0.4	4.6±0.5	4.7±0.4	4.8±0.5	4.7±0.3
HOMA-IR^1^	CID1	2.0±1.3	2.4±1.8	2.4±1.7	3.8±3.0	1.8±0.7	1.7±0.8	1.6±0.7	1.9±0.8	2.3±1.8	2.1±0.7
	CID2	1.2±0.9	1.4±0.8	1.3±0.9	1.8±1.2	1.0±0.6	1.1±0.4	1.1±0.6	1.1±0.5	1.5±1.3	1.5±0.5

Values are mean±SD from the fasted state. CID1: baseline, CID2: after 8-week weight loss; CID3: after 6-month weight maintenance/diet intervention. Bold values are different between continued weight-losers (WL) and weight-regainers (WR) by t-test (p<0.05), beside HOMR-IR by Mann-Whitney test.

1 Homeostasis model assessment of insulin resistance (HOMA-IR) was calculated as fasting glucose (mM)×fasting insulin (µIU/mL)/22.5.

According to the post-intervention dietary record (n = 73), the protein content was 17.8±4.1 and 20.7±5.4 energy% for LP and HP diets, respectively, and the GI was 56.0±4.6 and 59.5±4.5 for LGI and HGI diets, respectively. There was a modest but significant difference in dietary protein (p = 0.012) and in GI (p = 0.002) between the assigned dietary groups. The difference were confirmed by the urinary nitrogen excretion as a marker of adherence to HP or LP diet (13.8±3.3 and 11.8±3.3 g/day, p = 0.023).

### Logistic regression analysis to find predictors

We measured 31 blood proteins and 3 steroid hormones of 96 subjects at two time points before the dietary intervention/weight maintenance, namely at CID1 and CID2 (**Table S1**). Together with the fold changes during weight loss, these variables were analyzed by logistic regression to look for predictors of weight change during maintenance.

**Figure 1:**
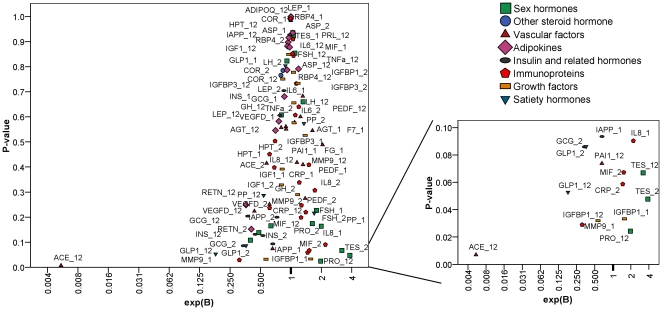
The predicting power of blood analytes for weight loss maintenance by Logistic Regression. Volcano plot of the significance P-value versus odd ratio exp(B) of blood proteins/steroids for predicting continued weight loss during the 6-month maintenance period by logistic regression controlled for age and the fold change of weight. The symbols of the analytes are listed in Table 1. Suffix “_1”: concentration at CID1, “_2”: concentration at CID2, “_12”: the fold change of concentration CID2/CID1. The analytes are grouped as in Table 1 and marked in different shape/color.

In the pooled subjects, the fold change of angiotensin I converting enzyme 1 (ACE, p = 0.007), progesterone (PRO, p = 0.024), IGF binding protein 1 (IGFBP1, p = 0.032), the baseline concentrations of MMP9 (p = 0.029) and IGFBP1 (p = 0.033), and the concentration of testosterone (TES, p = 0.048) at CID2 were significant to predict the outcome of weight maintenance. In addition, the baseline concentrations of IL8 (p = 0.090) and IAPP (p = 0.093), the concentrations of CRP (p = 0.059), macrophage migration inhibiting factor (MIF, p = 0.067), glucagon-like peptide-1 (GLP1, p = 0.086) and glucagon (GCG, p = 0.086) at CID2, and the fold change of GLP1 (p = 0.053), TES (p = 0.063) and plasminogen activator inhibitor-1 (PAI1, p = 0.074) had a trend towards significance (**Figure** 1).

When these 15 variables were analyzed by multinomial logistic regression, 8 were selected as most important independent variables to build up a prediction model (**Table** 3 Model 2). This model can correctly predict 83% of the cases, thus highly increased the prediction accuracy as compared to 59% with a model using only age and the fold change of weight (**Table** 3 Model 1).

**Table 3:** Logistic regression models to predict continued weight loss during the 6-month maintenance period.

Model		Parameters		
pseudo R^2^ (Nagelkerke)	model fitting p-value by Likelihood Ratio Tests	Variable	p-value by Wald Tests	Exp(B)
**1. Basal model**		Intercept	**0.020**	
0.108	0.017	age	0.111	0.93
		weight_12	**0.043**	5.3E-09
**2. Model without interactions**		Intercept	0.059	
0.653	1.5E-09	age	0.528	0.95
		weight_12	0.375	3.1E-06
		ACE_12	**<0.001**	3.0E-05
		MMP9_1	**0.002**	0.07
		TES_2	**0.006**	22.6
		IGFBP1_12	**0.007**	0.25
		MIF_2	**0.015**	2.40
		PAI1_12	**0.016**	0.40
		CRP_2	**0.034**	2.10
		IAPP_1	0.052	0.50
**3. Model with only interactions**		Intercept	**0.050**	
0.468	1.7E-04	[dietprotein = low]	**0.011**	1.6E+06
		[dietGI = low]	0.269	1.94
		age	**0.006**	0.85
		R_Weight_12	0.055	1.8E-11
		[dietGI = low] * IL8_12	**0.012**	0.03
		IL8_12	0.133	3.08
		[dietprotein = low] * LEP_1	**0.012**	0.02
		LEP_1	0.232	2.68
		[dietprotein = low] * LH_1	**0.035**	11.3
		LH_1	0.090	0.23
		[dietGI = low] * F7_1	0.061	115
		F7_1	0.218	0.13
		[dietGI = low] * MMP9_12	0.068	12.4
		MMP9_12	0.543	0.56
**4. Combined model**		Intercept	**0.014**	
0.835	3.2E-12	[dietprotein = low]	0.129	3.6E+07
		[dietGI = low]	0.483	2.46
		age	0.051	0.80
		Weight_12	0.122	4.2E-20
		ACE_12	**<0.001**	3.6E-11
		MMP9_1	**0.005**	0.03
		CRP_2	**0.007**	6.69
		PAI1_12	0.054	0.33
		TES_2	0.072	49.4
		IAPP_1	0.079	0.29
		[dietGI = low] *IL8_12	**0.001**	9.6E-06
		IL8_12	**0.030**	24.8
		[dietprotein = low] * LH_1	**0.010**	5.5E+03
		LH_1	**0.007**	2.1E-03
		[dietprotein = low] * LEP_1	0.145	0.01
		LEP_1	0.190	0.12

Dependent Variable is weight loss vs. weight regain during dietary intervention/maintenance.

The variables of measured blood analytes were Ln-transformed in the model. Suffix “_1”: concentration at CID1, “_2”: concentration at CID2, “_12”: fold change of the concentration (CID2/CID1). The symbol of blood analytes are listed in **Table** 1. In model 3 and 4, high dietary protein level and high GI level were reference categories and their related B = 0.

### RF to find predictors

**Figure 2:**
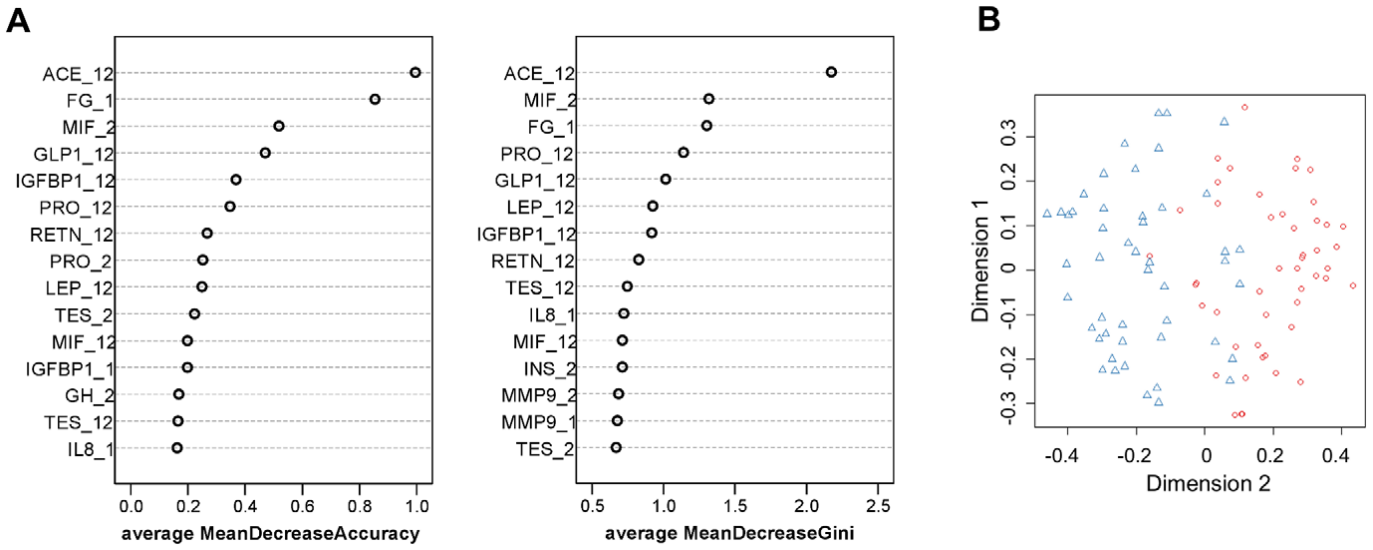
Top 15 important predictors for weight loss maintenance identified by Random Forest. **A**. The variables are ranked by the average of 10 runs on the mean decrease in classification accuracy (MDA) or by the mean decrease in classification Gini impurity (MDG). Suffix “_1”: concentration at CID1, “_2”: concentration at CID2, “_12”: fold change of the concentration (CID2/CID1). The symbol of blood analytes are listed in **Table** 1. **B**. Classification plot of continued weight-losers (red dots) and weight-regainers (blue triangles) during weight maintenance in pooled subjects (n = 96) by the top15 important variables.

All 98 blood protein/steroid variables were ranked by their MDG and MDA for the importance to classify the subjects into weight-losers or weight-regainers. With the pooled subjects the top 15 most important variables were identified based on MDG, which is very constant during classification permutation (**Figure 2**A). An optimal classification was achieved using this set of 15 variables, with an error rate of 24±1% (**Figure 2**B).

Overall, there is a strong correlation between the p-value from the logistic regression assay and the MDG from RF assay (r = 0.611, p<0.001 in Pearson correlation on Ln-transformed values). Nine of the top 15 most important variables from RF overlapped with significant or tending to be significant variables from logistic regression. The fold change of ACE was identified as the most important variable by both methods. For the top 5, only baseline FG is different from the logistic regression outcome. Its importance is even clearer when MDA is used for ranking.

**Figure 3:**
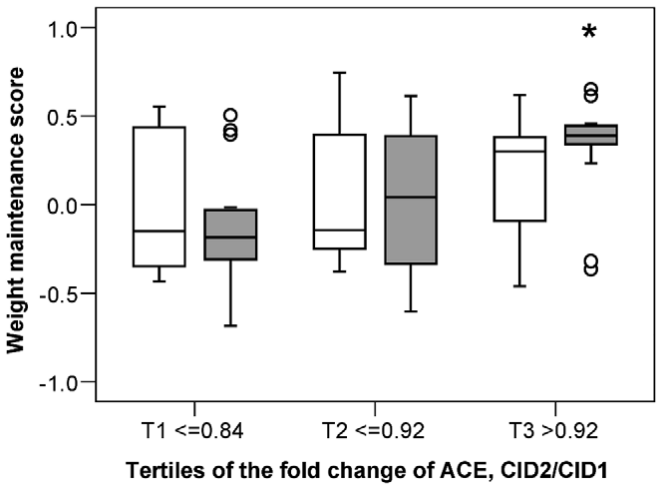
The relation between weight maintenance score and the fold change of ACE during weight loss. Boxplot shows the quartile range of weight maintenance score with outliers (in circle) across tertile of the fold change of ACE during weight loss, for subjects with low (≤9.6µmol/L, n = 48, blank bar) and with high (>9.6 µmol/L n = 47, grey bar) baseline fibrinogen level. The variation of weight maintenance score attributed to the fold change of ACE, p = 0.478 in low group and p = 0.014 in high group, was tested by one-way ANOVA controlled for age and the fold change of weight, and Bonferroni test for multiple comparisons. *T3 significantly different from T1 in high fibrinogen group, p = 0.013.

Because the interactions among variables increase their importance during making the decision trees, we checked possible interactions of baseline FG with other variables by logistic regression and found that it significantly interacts with the fold change of ACE on the prediction (p = 0.023). This interaction was still significant after controlling for the fold change of body fat mass (p = 0.014) as tested in a subset of subjects who had fat mass measured (n = 59). Based on the median baseline FG values we split the subjects into a low and a high group. Only in the high FG group was the fold change of ACE significantly associated with the outcome of weight maintenance, with a greater reduction in ACE predicting a greater chance for continued weight loss. In the low FG group, no difference was observed (**Figure 3**).

### Interaction with dietary protein and GI levels

**Figure 4:**
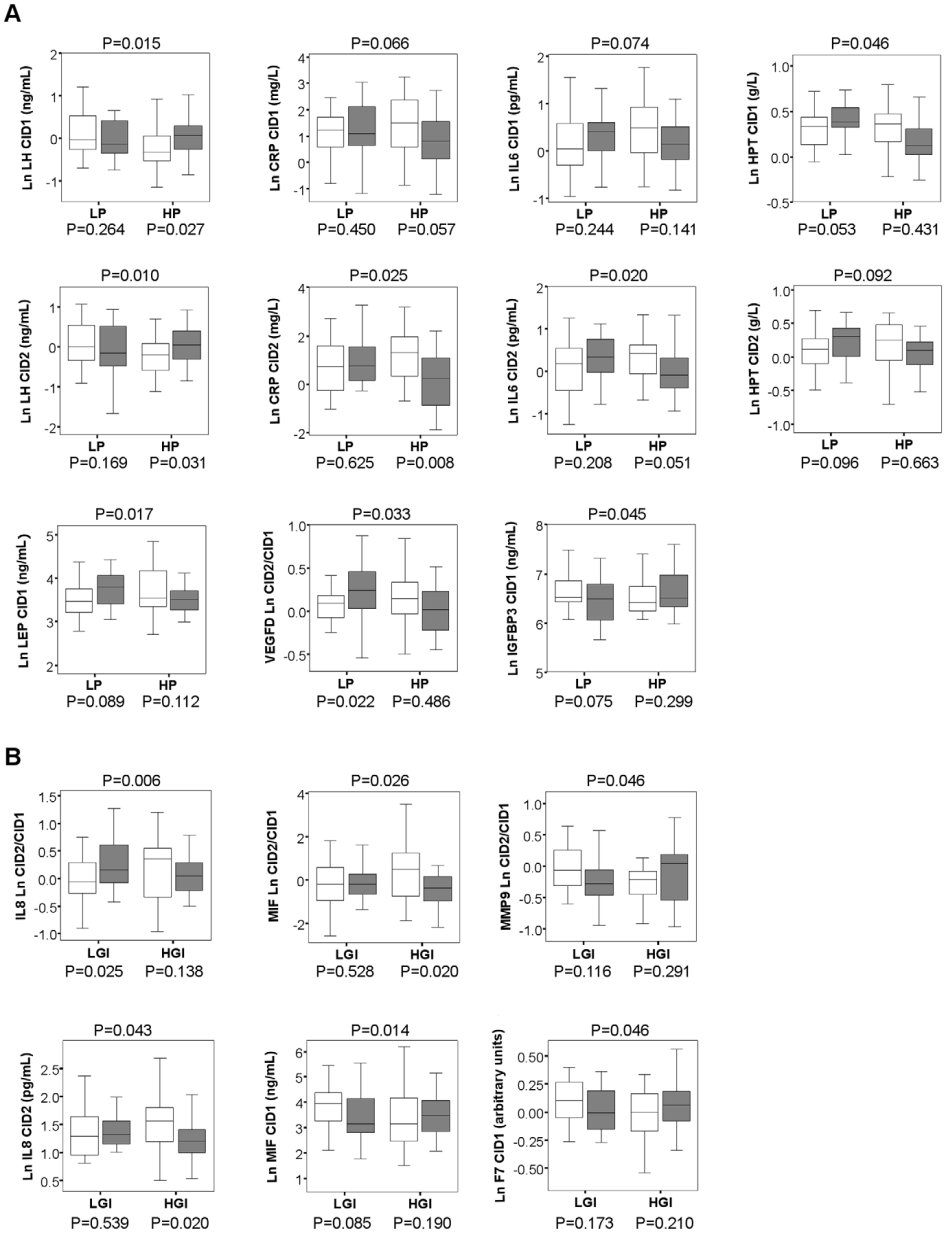
Predictors having interaction with dietary components for the outcome of weight maintenance. Boxplots show the quartile range of the blood analytes without outliers for continued weight-losers (blank bar) and weight-regainers (grey bar) in each dietary group. The p-value above the chart is the significance of the interaction between dietary protein/GI and the concentration/change of the blood analyte with respect to the outcome of weight maintenance (weight-loss or -regain). The p-values under the chart is the significance of the prediction of the variable inside the subgroups. All were obtained by logistic regression (controlled for age and the fold change of weight). **A**. predictors having interaction with dietary protein levels. LP: low protein, HP: high protein. **B**. predictors having interaction with dietary glycemic index (GI) levels. LGI: low GI, HGI: high GI.

By logistic regression, luteinizing hormone (LH), CRP, IL6, HPT, leptin (LEP), vascular endothelial growth factor-D (VEGFD) and IGFBP3 showed significant interaction with dietary protein level for the prediction of the weight change during maintenance (**Figure 4**A). Remarkably, immunoproteins CRP, IL6 and HPT showed the interaction either significantly or close to significant at both CID1 and CID2, and with the same pattern. They were also all positively correlated with LEP (p<0.002).

IL8, MIF, MMP9 and F7 showed significant interaction with dietary GI level for the prediction (**Figure 4**B). Among these analytes, IL8 and MIF were positively correlated (p<0.001).

A model using the independent interactions with dietary protein or GI (**Table** 3 Model 3) can correctly predict 77% of the cases. Multinomial logistic regression based on all components of model 2 and 3 resulted in a combined model with 9 independent analytes (**Table** 3 Model 4). This combined model can correctly predict 92% of the cases.

We also tried to use RF to search for the interactions between blood analytes and diet components on the prediction. However, there was no difference for the MDG of variables after taking the dietary components into the classification forests (p>0.99). This might be due to the fact that category variables of diet protein and GI were underrepresented by RF as compared to continuous variables [17].

### Correlations with ACE

Because ACE was identified as the most important predictor among all candidates, we checked for its relation with measures of obesity and other blood analytes (**Table** 4). It showed that ACE positively correlated to body weight (p = 0.036), body mass index (BMI, p = 0.024) and fat mass (p = 0.007) at baseline, but not significant anymore after weight loss. However, the contribution of the fold change of ACE to the prediction was still significant (p = 0.018) after controlling for baseline body fat mass in a subset of the cohort (n = 78), or close to significant (p = 0.086) after controlling for the fold change of fat mass (n = 60), while fat mass itself had no effect on the prediction (p>0.5).

**Table 4:** Significant correlations between ACE and measures of obesity and other blood analytes.

		Length^1^	CID1		CID2		CID2/CID1	
Category	Analyte	(AA)	*r*	p-value	*r*	p-value	*r*	p-value
Body adiposity	BMI	-	0.231	**0.024**	0.131	0.203	0.186	0.070
	weight	-	0.215	**0.036**	0.099	0.337	0.186	0.070
	Fat mass (%)^2^	-	0.304	**0.007**	0.202	0.086	−0.116	0.376
Sex hormones	FSH	92/111	0.198	0.053	0.024	0.813	0.463	**2.0E-06**
	LH	92/121	0.282	**0.005**	0.198	0.053	0.348	**5.2E-04**
	PRL	199	0.217	**0.035**	0.192	0.063	0.396	**7.1E-05**
Satiety factors	PP	36	0.220	**0.031**	0.251	**0.014**	0.263	**0.010**
	GLP1	37	0.196	0.056	0.135	0.189	0.329	**0.001**
Insulin related hormones	GCG	29	0.242	**0.017**	0.184	0.073	0.320	**0.002**
	IAPP	37	0.156	0.136	0.034	0.750	0.403	**8.1E-05**
Others	LEP	146	0.219	**0.032**	0.055	0.592	0.377	**1.5E-04**
	IL6	183	0.156	0.129	−0.024	0.818	0.256	**0.012**
	VEGFD	117	−0.134	0.192	−0.115	0.266	−0.289	**0.005**
	PAI1	379	0.242	**0.018**	0.017	0.870	0.130	0.205

Analyzed by Pearson correlation. Data of blood analytes were Ln transformed before analysis. *r*: correlation coefficient, *p*: significance. The significant p-values (<0.05) are marked as bold.

1 The length of the (main) active processed chain is resourced from the UniProt Knowledgebase (http://beta.uniprot.org/uniprot/).

2 Subjects used in the analysis with respect to fat mass measured at CID1 n = 78, at CID1 n = 73, and the fold change CID2/CID1 n = 60. For other analytes subjects n = 96.

All measured peptide sex hormones, satiety hormones and insulin related hormones, but not insulin itself, were positively correlated with ACE. For most proteins this correlation concerned their fold changes. Except for PAI1, all those factors are low molecular weight proteins/peptides.

## Discussion

### An important role of ACE in weight maintenance

ACE is a zinc metallopeptidase and catalyses the hydrolysis of dipeptides or tripeptides from the carboxyl terminus of oligopeptides [18]. Its most well-known product is angiotensin II from the substrate angiotensinogen, forming the renin-angiotensin system, which contributes to increased blood pressure and retains salt and water [19]. The genetic polymorphisms in this gene, which highly influence the ACE circulating level [20], have been repeatedly found to be associated with measures of obesity [6]. Our data support this. We observed that moderate weight loss by LCD significantly decreased ACE concentration about 12%, which is in line with previously reported decreased ACE activity in overweight/obese woman (about 12%) [21], and in mixed male and female obese adults (about 20%) by weight loss [22]. The relation between ACE and obesity has been suggested to lie in the local expression of the renin-angiotensin system and the possible trophic role of angiotensin II in the development of adipose tissue [23], [24]. Moreover, its role in water retention may also contribute to regulation of body weight gain.

In the present study we showed correlations between ACE and sex hormones, satiety hormones, insulin related hormones, LEP and other blood metabolic proteins, suggesting its broad range of substrates and involved pathways. Indeed, ACE was found to be able to process gonadotropin-releasing hormone (LHRH) in vitro, thus possibly regulating both LH and follicle-stimulating hormone levels [25]. Also satiety hormones and GCG need to be proteolytically processed to become active [26], [27]. But it is yet unknown if ACE plays a role in this process or not. Moreover, the prediction by ACE was independent of body weight and fat mass. Thus we speculate that the role of ACE in obesity development and weight regain touches a complex network, not only involving adipose tissue development, but also water and sodium retention, and possibly satiety hormone regulation to control energy intake. It is further known that ACE is abundant in the hypothalamus [28]. A recent observation on the crosstalk between ACE and uncoupling protein-2 (UCP2) in human umbilical vein endothelial cells [29] may support this, because UCP2 in the hypothalamus regulates the function of neurons involved in food intake during fasting [30].

Here for the first time, we found that neither the baseline level, nor the post-weight-loss level, but the extent of the reduction of ACE during weight loss discriminates between subjects who will continue to lose weight and those who will regain weight during the 6-month weight maintenance period. A greater reduction in ACE predicts a greater chance for continued weight loss. However, the predicting power of the fold change of ACE alone is limited due to a large overlap between weight-losers and -regainers. Apparently, more processes in the body are involved in weight regain/maintenance than the range of pathways that ACE may cover.

We also reported a novel interaction between ACE and FG, namely the prediction power of ACE is only observed in subjects with high baseline FG. FG is the key component of blood coagulation, but it is also an anti-inflammatory acute phase protein [31] and can serve as a biomarker for obesity [32]. Among other inflammation-related proteins, baseline levels of IL6, MMP9, MIF and HPT were also shown to interact with the fold change of ACE (p = 0.003, 0.026, 0.028 and 0.028, respectively) with the same pattern as FG, but IL8, CRP and TNFα did not. While the induction of factors like CRP requires various signals including TNFα, induction of FG only requires IL6 [31]. This suggests that an IL6-mediated inflammation state may amplify the role of ACE in weight maintenance.

### Interactions between blood analytes and dietary components

Our findings show that the prediction can be manipulated by the dietary protein and GI intake during the weight maintenance period. We did not perform multiple testing corrections, but the repeatedly detected similar interaction of analytes from the same functional group may secure the finding. This is the case for the interaction between dietary protein level and immunoproteins (IL6, CRP and HPT). The interactions between GI level and other immunoproteins IL8, MIF and MMP9 were not consistent. Therefore we only discuss the role of dietary protein in weight maintenance. Our results suggest that in order to prevent weight regain, subjects with a high baseline level of LEP, IL6, CRP and HPT should follow a HP diet, and subjects with a low baseline level are most likely to succeed with a LP diet.

In the Diogenes study, the fat content was kept relatively constant among diets. As a consequence HP diets also mean low carbohydrate diets [3], [5]. Thus, the aforementioned interaction with dietary protein level might also be interpreted as the interaction with dietary carbohydrate level. During the weight maintenance after weight loss, adipocytes try to recover energy storage by increasing the uptake of glucose and fat [33]. But also immune responses are energy expensive processes and glucose is the preferred energy fuel [34], [35]. When there is a competition for glucose, the survival related immune system may have priority over the storage function of adipocytes. With LP (high carbohydrate) diets, there might be no energy competition and adipocytes can dominate the fuel flow. With HP (low carbohydrate) diets, there is energy competition and the immune system can dominate the fuel flow.

LEP represents the amount/size of adipocytes and/or activity of adipose tissue, confirmed by the strong correlation between LEP and fat mass at baseline in our study (r = 0.595, p<0.001). As expected, the interaction between LEP and dietary protein level lost significance (p = 0.190) if we add the interaction between fat mass and dietary protein level (p = 0.418) in the model. Thus subjects with high LEP level taking LP diet will easily recover energy storage without fuel flow restriction. The relation between immunoproteins and weight regain may also be a secondary effect, because immune system and adipocytes/adipose tissue are positively associated, confirmed by strong correlations between LEP and immunoproteins in our study. Also their interactions with dietary protein were not independent from each other. For subjects with profound/active fat mass, a HP diet is preferred to prevent weight regain.

### Limitations and Conclusions

Because obesity and weight regulation is complex, a panel of predictors covering various processes performs better than one or two predictors on the prediction of weight change after weight loss. A logistic regression model with 9 independent predictors has an accuracy of prediction of >90%. However, such self evaluation is too optimistic. RF gave a more realistic evaluation with a moderate accuracy of 76% by a 15-predictor panel. The present study was conducted on a limited number of adult females extracted from a pan-European project. Therefore, our findings should be validated in other cohorts and also in males. Nevertheless, the information about 34 blood proteins/steroids, particularly the importance of ACE, and the interaction between dietary protein/carbohydrate level and LEP and immunoproteins, may help to develop personalized programmes to improve weight maintenance.

## Supporting Information

Text S1 Selection of analytes as predictor.
(DOC)

Table S1 Measured blood proteins and steroids by diet and the outcome of weight maintenance at baseline (CID1) and after 8-week weight loss (CID2).
(DOC)
