# Correction: Blood Profile of Proteins and Steroid Hormones Predicts Weight Change after Weight Loss with Interactions of Dietary Protein Level and Glycemic Index

**DOI:** 10.1371/annotation/2526dde5-cfaa-4f00-b554-25768bda2935

**Published:** 2011-07-13

**Authors:** Ping Wang, Claus Holst, Malene R. Andersen, Arne Astrup, Freek G. Bouwman, Sanne van Otterdijk, Will K. W. H. Wodzig, Marleen A. van Baak, Thomas M. Larsen, Susan A. Jebb, Anthony Kafatos, Andreas F. H. Pfeiffer, J. Alfredo Martinez, Teodora Handjieva-Darlenska, Marie Kunesova, Wim H. M. Saris, Edwin C. M. Mariman

The authors would like to add the following to the Competing Interests statement: "A patent application concerning part of the results of this study has been filed by the University Maastricht (Application no. 10172846.7-2401)."

