# Supplementary material for: Blood Profile of Proteins and Steroid Hormones Predicts Weight Change after Weight Loss with Interactions of Dietary Protein Level and Glycemic Index
Source: PLoS One. 2011 Feb 14;6(2):e16773. doi: 10.1371/journal.pone.0016773 (PMC3038864; doi:10.1371/journal.pone.0016773)
Supplement: Text S1 — Selection of analytes as predictor. (DOC) [file pone.0016773.s001.doc]

## Text S1. Selection of analytes as predictor candidates

PubMed was searched on obesity and dietary intervention with measurement of plasma proteins and peptides for potential candidates as predictor of weight maintenance. We focused on proteins that are linked to physiologic mechanisms of obesity.

The Human Obesity Gene Map reviewed annually all markers, genes and mutations associated or linked with obesity phenotypes until 2005. In the latest version, 22 genes were reported whose association with obesity-phenotypes have been confirmed by at least 5 positive studies [1,2]. Among these 22 genes, 7 genes encode plasma proteins: angiotensin I-converting enzyme 1 (ACE), adiponectin (ADIPOQ), interleukin 6 (IL6), insulin (INS), leptin (LEP), resistin (RETN) and tumor necrosis factor (TNF). In addition, based on a Diogenes study on obesity genes, matrix metalloproteinase 9 (MMP9) [3] and islet amyloid polypeptide (amylin, IAPP) [4] were included as candidates.

In addition to LEP, ADIPOQ and RETN, there are many other proteins secreted by adipocytes as adipokines. We selected retinal-binding protein 4 (RBP4) [5,6] and acylation stimulating protein (C3adesArg, ASP) [7,8], which have been reported to participate in the weight loss process.

Obesity is often associated with chronic inflammation [9,10]. In addition to cytokines IL6 and TNFa that have been confirmed by genetic studies, other chemokines and acute phase reactants, including interleukin 8 (IL8) [11], macrophage migration inhibitory factor (MIF) [12], C-reactive protein (CRP) [13], and haptoglobin (HPT) [14] were included in this study. Except CRP, the others are also expressed by adipocytes [15].

Obesity is also linked to thrombosis [16]. Plasminogen activator inhibitor 1 (PAI-1), of which the primary role in blood is to inhibit the activation of plasminogen [17], is also well recognised as an adipokine. Two proteins involved in blood coagulation that are also sensitive to inflammation and elevated in obesity, fibrinogen (FG) [18] and coagulation factor VII (F7) [19], were also included.

Angiogenesis is an essential process for adipose tissue development [20]. The (anti-)angiogenic factors related to obesity vascular endothelial growth factor-D (VEGFD) [21] and pigment epithelium-derived factor (PEDF) [22], were regarded as potential candidates.

## References

1. Rankinen T, Zuberi A, Chagnon YC, Weisnagel SJ, Argyropoulos G, et al. (2006) The human obesity gene map: the 2005 update. Obesity (Silver Spring) 14: 529-644.

2. Walley AJ, Blakemore AI, Froguel P (2006) Genetics of obesity and the prediction of risk for health. Hum Mol Genet 15 Spec No 2: R124-130.

3. Chavey C, Mari B, Monthouel MN, Bonnafous S, Anglard P, et al. (2003) Matrix metalloproteinases are differentially expressed in adipose tissue during obesity and modulate adipocyte differentiation. J Biol Chem 278: 11888-11896.

4. Arnelo U, Blevins JE, Larsson J, Permert J, Westermark P, et al. (1996) Effects of acute and chronic infusion of islet amyloid polypeptide on food intake in rats. Scand J Gastroenterol 31: 83-89.

5. Janke J, Engeli S, Boschmann M, Adams F, Bohnke J, et al. (2006) Retinol-binding protein 4 in human obesity. Diabetes 55: 2805-2810.

6. Vitkova M, Klimcakova E, Kovacikova M, Valle C, Moro C, et al. (2007) Plasma levels and adipose tissue messenger ribonucleic acid expression of retinol-binding protein 4 are reduced during calorie restriction in obese subjects but are not related to diet-induced changes in insulin sensitivity. J Clin Endocrinol Metab 92: 2330-2335.

7. Sniderman AD, Cianflone KM, Eckel RH (1991) Levels of acylation stimulating protein in obese women before and after moderate weight loss. Int J Obes 15: 333-336.

8. Cianflone K, Xia Z, Chen LY (2003) Critical review of acylation-stimulating protein physiology in humans and rodents. Biochim Biophys Acta 1609: 127-143.

9. Ferrante AW (2007) Obesity-induced inflammation: a metabolic dialogue in the language of inflammation. Journal of Internal Medicine 262: 408-414.

10. Das UN (2001) Is obesity an inflammatory condition? Nutrition 17: 953-966.

11. Bruun JM, Pedersen SB, Kristensen K, Richelsen B (2002) Opposite regulation of interleukin-8 and tumor necrosis factor-alpha by weight loss. Obes Res 10: 499-506.

12. Kleemann R, Bucala R Macrophage migration inhibitory factor: critical role in obesity, insulin resistance, and associated comorbidities. Mediators Inflamm 2010: 610479.

13. Rexrode KM, Pradhan A, Manson JE, Buring JE, Ridker PM (2003) Relationship of total and abdominal adiposity with CRP and IL-6 in women. Ann Epidemiol 13: 674-682.

14. Chiellini C, Santini F, Marsili A, Berti P, Bertacca A, et al. (2004) Serum haptoglobin: a novel marker of adiposity in humans. J Clin Endocrinol Metab 89: 2678-2683.

15. Fain JN (2006) Release of interleukins and other inflammatory cytokines by human adipose tissue is enhanced in obesity and primarily due to the nonfat cells. Vitam Horm 74: 443-477.

16. De Pergola G, Pannacciulli N (2002) Coagulation and fibrinolysis abnormalities in obesity. Journal of Endocrinological Investigation 25: 899-904.

17. Loskutoff DJ, Samad F (1998) The adipocyte and hemostatic balance in obesity: studies of PAI-1. Arterioscler Thromb Vasc Biol 18: 1-6.

18. Festa A, D'Agostino R, Jr., Williams K, Karter AJ, Mayer-Davis EJ, et al. (2001) The relation of body fat mass and distribution to markers of chronic inflammation. Int J Obes Relat Metab Disord 25: 1407-1415.

19. Reiner AP, Carlson CS, Rieder MJ, Siscovick DS, Liu K, et al. (2007) Coagulation factor VII gene haplotypes, obesity-related traits, and cardiovascular risk in young women. Journal of Thrombosis and Haemostasis 5: 42-49.

20. Rupnick MA, Panigrahy D, Zhang CY, Dallabrida SM, Lowell BB, et al. (2002) Adipose tissue mass can be regulated through the vasculature. Proc Natl Acad Sci U S A 99: 10730-10735.

21. Silha JV, Krsek M, Sucharda P, Murphy LJ (2005) Angiogenic factors are elevated in overweight and obese individuals. Int J Obes (Lond) 29: 1308-1314.

22. Wang P, Smit E, Brouwers M, Goossens GH, van der Kallen CJ, et al. (2008) Plasma pigment epithelium-derived factor is positively associated with obesity in Caucasian subjects, in particular with the visceral fat depot. European Journal of Endocrinology 159: 713-718.
