# Supplementary material for: Blood Profile of Proteins and Steroid Hormones Predicts Weight Change after Weight Loss with Interactions of Dietary Protein Level and Glycemic Index
Source: PLoS One. 2011 Feb 14;6(2):e16773. doi: 10.1371/journal.pone.0016773 (PMC3038864; doi:10.1371/journal.pone.0016773)
Supplement: Table S1 — Measured blood proteins and steroids by diet and the outcome of weight maintenance at baseline (CID1) and after 8-week weight loss (CID2). (DOC) [file pone.0016773.s002.doc]

## Table S1. Measured blood proteins and steroids by diet and the outcome of weight maintenance (WL: continued weight-losers, WR: weight-regainers ) at baseline (CID1) and after 8-week weight loss (CID2).

|  |  | pooled | | Diet1 LP/LGI | | Diet2 LP/HGI | | Diet3 HP/LGI | | Diet4 HP/HGI | |
| --- | --- | --- | --- | --- | --- | --- | --- | --- | --- | --- | --- |
| Analyte |  | WL N=48 | WR N=48 | WL N=12 | WR N=12 | WL  N=12 | WR N=12 | WL  N=12 | WR N=12 | WL N=12 | WR N=12 |
| Cortisol (ng·mL^-1^) | CID1 | 105 (78–129) | 99 (81–128) | 105 (92–115) | 123 (81–139) | 118 (85–133) | 94 (82–119) | 103 (71–152) | 89 (80–124) | 95 (75–114) | 97 (76–120) |
|  | CID2 | 102 (84–127) | 104 (88–132) | 93 (85–121) | 104 (98–140) | 120 (93–134) | 111 (101–134) | 107 (78–124) | 105 (86–132) | 89 (70–165) | 89 (67–111) |
| Progesterone (ng· mL^-1^) | CID1 | 5.8 (3.7–10.7) | 6.4 (4.6–9.5) | 5.7 (4.4–6.8) | 6.5 (5.1–9.2) | 5.8 (2.0–10.7) | 6.2 (5.2–8.7) | 4.7 (3.1–12.7) | 8.4 (4.3–15.0) | 8.1 (5.5–18.5) | 5.2 (3.5–8.9) |
|  | CID2 | 7.2 (4.1–10.0) | 5.9 (3.7–7.8) | 8.4 (5.3–15.3) | 5.7 (4.3–6.9) | 6.0 (4.6–11.0) | 7.2 (4.6–9.0) | 7.9 (3.8–11.5) | 4.3 (3.1–6.9) | 5.5 (3.6–9.5) | 6.5 (2.8–9.2) |
| Testosterone (ng· mL^-1^) | CID1 | 0.83 (0.64–1.00) | 0.78 (0.62–1.10) | 0.94 (0.64–1.08) | 0.80 (0.75–0.98) | 0.66 (0.51–1.08) | 0.82 (0.47–1.13) | 0.80 (0.64–0.97) | 0.65 (0.49–0.93) | 0.88 (0.79–0.98) | 1.10 (0.76–1.20) |
|  | CID2 | 0.86 (0.69–1.10) | 0.77 (0.61–0.89) | 0.86 (0.70–1.14) | 0.79 (0.64–0.89) | 0.88 (0.62–1.20) | 0.77 (0.41–0.86) | 0.80 (0.71–0.93) | 0.74 (0.54–0.87) | 0.99 (0.65–1.28) | 0.87 (0.61–1.18) |
| FSH (ng· mL^-1^) | CID1 | 9.0 (7.5–12.0) | 8.7 (6.2–11.0) | 10.2 (7.3–12.8) | 8.4 (5.9–13.0) | 9.2 (7.5–12.0) | 7.8 (5.4–11.0) | 8.2 (6.2–11.8) | 8.5 (5.7–9.9) | 8.8 (8.1–11.8) | 10.5 (7.7–18.5) |
|  | CID2 | 9.2 (7.5–12.0) | 9.3 (6.7–13.0) | 9.4 (7.2–16.5) | 7.5 (6.5–9.1) | 9.3 (7.0–17.0) | 8.9 (5.5–12.5) | 8.8 (7.5–14.0) | 11.0 (8.0–12.8) | 9.1 (8.0–11.8) | 9.8 (5.8–15.8) |
| LH (ng· mL^-1^) | CID1 | 0.83 (0.67–1.40) | 0.98 (0.73–1.40) | 0.96 (0.80–1.58) | 0.93 (0.60–1.58) | 0.92 (0.70–2.03) | 0.84 (0.70–1.25) | 0.76 (0.55–1.30) | 1.10 (0.55–1.40) | 0.71 (0.58–1.06) | 1.05 (0.78–1.30) |
|  | CID2 | 0.91 (0.64–1.48) | 0.94 (0.65–1.58) | 1.10 (0.78–1.95) | 0.93 (0.54–1.78) | 1.02 (0.70–1.58) | 0.70 (0.62–1.63) | 0.96 (0.65–1.18) | 1.15 (0.85–1.50) | 0.71 (0.53–0.94) | 0.97 (0.68–1.60) |
| PRL (ng· mL^-1^) | CID1 | 14.0 (9.8–18.0) | 13.0 (10.0–17.0) | 13.0 (8.1–18.8) | 12.0 (9.3–23.0) | 11.5 (8.7–14.0) | 13.0 (12.0–17.0) | 13.5 (11.0–14.0) | 16.0 (11.5–18.5) | 16.5 (10.7–19.5) | 13.0 (10.5–15.8) |
|  | CID2 | 13.0 (9.8–16.8) | 13.0 (12.0–17.0) | 12.0 (9.7–14.5) | 12.0 (10.0–14.0) | 12.0 (10.0–15.0) | 12.0 (12.0–17.0) | 13.5 (9.1–17.8) | 16.5 (13.5–19.5) | 13.0 (11.3–18.5) | 13.5 (10.5–17.8) |
| ACE (ng· mL^-1^) | CID1 | 166 (117–197) | 146 (118–186) | 173 (129–216) | 184 (123–217) | 144 (97–183) | 136 (110–157) | 167 (102–197) | 168 (122–201) | 168 (118–197) | 126 (94–172) |
|  | CID2 | 134 (95–168) | 135 (114–166) | 151 (110–175) | 146 (115–187) | 125 (79–167) | 121 (106–136) | 133 (89–155) | 147 (126–166) | 127 (103–183) | 122 (96–165) |
| AGT (ng· mL^-1^) | CID1 | 7.3 (3.0–14.8) | 6.8 (2.4–17.0) | 5.6 (2.5–12.8) | 5.0 (3.2–11.8) | 4.1 (2.5–13.8) | 7.1 (3.9–24.8) | 9.7 (4.6–51.0) | 5.0 (1.4–24.0) | 9.4 (3.3–46.0) | 7.1 (1.9–16.0) |
|  | CID2 | 5.1 (2.3–19.8) | 6.4 (1.8–16.8) | 4.0 (2.1–19.4) | 5.9 (2.3–11.5) | 6.3 (2.3–13.5) | 10.3 (1.7–20.8) | 16.5 (3.0–52.0) | 9.2 (2.0–18.8) | 6.7 (2.2–13.5) | 3.7 (1.6–23.8) |
| F7 (arbitrary units) | CID1 | 1.0 (0.9–1.3) | 1.0 (0.9–1.2) | 1.1 (0.9–1.3) | 1.0 (0.8–1.3) | 1.1 (0.7–1.2) | 1.1 (0.9–1.3) | 1.2 (1.0–1.4) | 1.0 (0.9–1.1) | 1.0 (0.9–1.1) | 1.0 (0.9–1.2) |
| FG (µmol· L^-1^) | CID1 | 9.7 (8.4–11.4) | 9.4 (7.6–10.9) | 8.8 (8.2–9.7) | 10.0 (7.7–11.5) | 9.2 (8.4–11.0) | 9.9 (8.0–11.5) | 9.8 (8.3–11.8) | 9.6 (7.9–10.5) | 10.1 (8.5–11.9) | 8.1 (6.6–9.8) |
| PAI1 (ng· mL^-1^) | CID1 | 2.7 (0.9–6.3) | 2.4 (1.1–4.3) | 3.7 (1.5–7.1) | 3.5 (1.1–5.4) | 1.5 (0.5–6.7) | 2.4 (0.7–2.9) | 1.8 (0.6–3.6) | 2.1 (0.2–4.8) | 4.9 (1.0–6.9) | 3.4 (1.2–5.9) |
|  | CID2 | 1.0 (0.5–2.6) | 1.4 (0.8–2.9) | 1.1 (0.4–2.7) | 1.8 (0.9–4.6) | 0.7 (0.6–3.3) | 0.8 (0.3–1.8) | 1.1 (0.5–2.6) | 1.7 (0.8–2.9) | 1.3 (0.5–3.1) | 2.0 (1.1–3.7) |
| ADIPOQ (µg· mL^-1^) | CID1 | 2.6 (2.0–3.0) | 2.6 (1.8–3.3) | 2.6 (2.0–3.0) | 2.0 (1.4–2.5) | 2.2 (1.8–3.4) | 2.7 (2.2–4.1) | 2.6 (2.1–2.8) | 3.2 (2.4–3.7) | 2.6 (2.4–3.4) | 1.9 (1.7–3.0) |
|  | CID2 | 2.7 (2.0–3.4) | 2.6 (2.0–3.5) | 2.8 (2.0–3.5) | 2.1 (1.5–2.7) | 2.2 (1.8–3.5) | 2.7 (2.2–4.2) | 2.8 (2.2–3.5) | 3.1 (2.4–4.3) | 2.8 (2.0–3.4) | 2.7 (1.7–3.4) |
| ASP (mg· mL^-1^) | CID1 | 3.3 (2.5–4.5) | 3.3 (2.1–4.5) | 3.8 (2.2–4.6) | 3.3 (2.2–4.8) | 3.1 (2.1–3.7) | 3.5 (2.5–4.5) | 3.3 (1.5–4.9) | 2.8 (1.9–5.6) | 4.2 (2.8–5.4) | 3.8 (2.0–4.3) |
|  | CID2 | 3.4 (2.7–4.1) | 3.4 (2.2–4.2) | 3.2 (1.8–4.1) | 3.2 (2.1–4.2) | 3.1 (2.1–3.8) | 3.5 (2.5–4.5) | 3.5 (1.7–4.1) | 3.5 (1.8–4.1) | 3.7 (3.4–4.4) | 3.4 (2.0–4.2) |
| LEP (ng· mL^-1^) | CID1 | 32.5 (26.3–51.0) | 37.0 (29.3–47.8) | 36.0 (27.5–46.5) | 43.0 (31.0–54.0) | 31.5 (25.0–41.5) | 44.5 (29.3–62.3) | 39.0 (30.3–66.8) | 31.0 (25.3–35.8) | 31.0 (26.3–61.3) | 37.0 (23.3–42.5) |
|  | CID2 | 13.0 (9.3–21.0) | 18.5 (11.5–27.0) | 19.0 (12.0–21.0) | 20.0 (15.3–29.8) | 11.5 (6.1–18.8) | 19.5 (11.8–29.8) | 13.5 (12.0–21.5) | 11.5 (7.6–23.8) | 9.6 (7.3–27.5) | 18.5 (13.8–28.5) |
| RBP4 (µg· mL^-1^) | CID1 | 25.3 (12.4–87.3) | 28.3 (13.5–77.6) | 24.6 (9.4–82.7) | 24.8 (17.0–106.0) | 58.4 (12.7–118.0) | 61.7 (26.4–163.2) | 19.2 (12.2–64.8) | 17.7 (12.0–26.9) | 46.2 (14.9–88.6) | 31.1 (9.8–73.6) |
|  | CID2 | 37.2 (10.3–92.6) | 28.1 (13.0–92.2) | 51.2 (8.4–92.6) | 35.8 (13.5–100.7) | 36.7 (10.7–54.4) | 45.9 (18.8–101.1) | 14.0 (9.1–108.9) | 14.9 (10.0–36.6) | 50.5 (11.5–128.4) | 23.9 (9.1–92.2) |
| RETN (ng· mL^-1^) | CID1 | 3.8 (3.0–5.0) | 3.8 (3.1–5.0) | 3.6 (2.6–4.6) | 4.4 (3.2–6.2) | 3.9 (3.0–6.5) | 4.2 (3.3–5.0) | 3.5 (3.1–4.9) | 3.4 (2.8–3.9) | 4.5 (3.4–5.4) | 4.0 (3.0–5.6) |
|  | CID2 | 3.8 (3.1–5.1) | 4.1 (3.3–4.9) | 3.8 (2.9–5.6) | 4.6 (3.3–5.7) | 3.6 (3.1–4.5) | 4.6 (3.6–6.5) | 4.0 (2.8–6.0) | 4.1 (3.4–4.9) | 4.5 (3.5–5.1) | 3.8 (3.1–4.2) |
| GCG (pg· mL^-1^) | CID1 | 991 (756–1193) | 937 (736–1398) | 1070 (776–1268) | 904 (759–1330) | 1010 (786–1413) | 964 (567–1545) | 1028 (641–1233) | 883 (672–1155) | 920 (742–987) | 941 (691–1463) |
|  | CID2 | 913 (768–1088) | 930 (804–1320) | 928 (712–1073) | 918 (812–1255) | 956 (764–1318) | 856 (467–1358) | 930 (781–1293) | 1040 (783–1205) | 871 (667–1011) | 951 (867–1413) |
| IAPP (pmol· L^-1^) | CID1 | 5.7 (4.4–9.9) | 7.3 (5.4–10.9) | 5.8 (3.8–6.5) | 10.1 (6.0–12.5) | 5.4 (2.6–6.7) | 6.7 (3.0–10.5) | 6.9 (4.6–10.7) | 6.6 (4.4–9.5) | 9.0 (4.6–19.1) | 8.1 (5.7–19.1) |
|  | CID2 | 4.1 (2.2–7.4) | 5.0 (3.4–8.4) | 3.5 (2.2–6.4) | 4.9 (3.8–8.9) | 3.4 (1.3–4.8) | 4.4 (2.6–6.2) | 5.6 (2.4–16.6) | 4.6 (2.5–7.3) | 6.9 (2.7–8.5) | 7.4 (4.7–14.9) |
| Insulin  (µIU· mL^-1^) | CID1 | 9.1 ± 4.9 | 10.2 ± 6.7 | 10.0 ± 5.5 | 15.1 ± 10.8 | 8.5 ± 3.5 | 8.1 ± 3.7 | 7.5 ± 2.3 | 8.0 ± 3.9 | 10.5 ± 7.1 | 9.5 ± 3.1 |
|  | CID2 | 5.7 ± 3.6 | 6.5 ± 3.0 | 5.6 ± 3.6 | 7.9 ± 4.6 | 4.9 ± 2.8 | 5.5 ± 1.7 | 5.7 ± 3.1 | 5.4 ± 2.2 | 6.8 ± 4.8 | 7.4 ± 2.2 |
| CRP (µg· mL^-1^) | CID1 | 3.7 (1.7–7.1) | 2.8 (1.4–6.4) | 4.3 (1.7–6.6) | 6.8 (1.9–10.7) | 2.0 (1.7–3.7) | 2.6 (1.4–6.1) | 4.5 (2.4–12.0) | 2.3 (0.8–5.5) | 4.2 (1.4–15.8) | 2.2 (1.3–4.7) |
|  | CID2 | 2.4 (1.2–5.6) | 1.7 (0.8–4.6) | 1.9 (1.2–3.2) | 2.1 (1.1–4.8) | 2.3 (0.5–5.4) | 1.9 (1.0–4.9) | 4.2 (1.5–5.8) | 1.5 (0.4–5.1) | 2.1 (1.4–11.1) | 1.3 (0.5–2.5) |
| IL6 (pg· mL^-1^) | CID1 | 1.4 (0.8–2.2) | 1.3 (0.9–1.8) | 1.3 (0.9–2.1) | 1.7 (1.2–1.9) | 0.9 (0.7–1.6) | 1.4 (0.8–1.6) | 1.6 (0.9–3.0) | 1.2 (0.9–1.6) | 1.7 (1.0–2.4) | 1.3 (0.6–2.0) |
|  | CID2 | 1.3 (0.8–1.8) | 1.1 (0.8–1.8) | 1.3 (0.7–1.6) | 1.3 (1.0–2.2) | 0.9 (0.5–1.9) | 1.6 (0.9–2.1) | 1.6 (0.9–1.9) | 1.1 (0.7–1.5) | 1.4 (0.9–1.9) | 0.8 (0.6–1.3) |
| IL8 (pg· mL^-1^) | CID1 | 3.8 (2.8–5.3) | 3.4 (2.1–4.7) | 3.6 (2.8–5.4) | 3.7 (1.9–4.8) | 3.2 (2.1–7.4) | 3.2 (1.6–4.0) | 4.4 (2.7–5.3) | 3.2 (1.7–3.9) | 3.8 (3.0–4.6) | 4.0 (2.7–5.9) |
|  | CID2 | 4.1 (3.0–5.6) | 3.5 (3.0–4.7) | 2.9 (2.5–4.9) | 4.1 (3.2–5.0) | 5.5 (3.6–6.7) | 3.4 (2.9–4.0) | 4.2 (3.0–5.3) | 3.5 (3.2–4.6) | 4.0 (3.1–5.4) | 3.2 (2.6–6.0) |
| HP (g· L^-1^) | CID1 | 1.4 (1.1–1.6) | 1.4 (1.1–1.6) | 1.4 (1.1–1.6) | 1.7 (1.5–1.8) | 1.4 (1.2–1.5) | 1.4 (1.1–1.5) | 1.4 (1.4–1.5) | 1.1 (1.0–1.3) | 1.4 (0.8–1.8) | 1.2 (1.1–1.5) |
|  | CID2 | 1.2 (0.9–1.5) | 1.2 (1.0–1.4) | 1.0 (0.9–1.1) | 1.4 (1.2–1.7) | 1.2 (1.1–1.4) | 1.2 (0.9–1.4) | 1.4 (1.0–1.7) | 1.0 (0.6–1.3) | 1.1 (0.5–1.5) | 1.1 (1.1–1.3) |
| MIF (ng· mL^-1^) | CID1 | 29.5 (17.4–78.9) | 25.7 (16.5–60.7) | 58.6 (23.7–148.0) | 22.0 (8.8–125.3) | 11.6 (8.8–32.2) | 32.9 (17.7–54.8) | 51.9 (26.8–77.3) | 23.9 (16.6–53.9) | 36.7 (21.0–97.2) | 31.9 (14.4–131.3) |
|  | CID2 | 52.2 (17.0–102.0) | 22.9 (13.6–44.9) | 57.4 (20.6–100.7) | 25.0 (16.0–98.1) | 56.9 (16.1–127.8) | 22.2 (15.1–30.1) | 45.1 (15.2–101.0) | 20.2 (13.1–35.4) | 57.8 (18.1–91.2) | 28.5 (11.7–71.3) |
| MMP9 (ng· mL^-1^) | CID1 | 422 (283–525) | 456 (347–603) | 445 (245–533) | 530 (430–647) | 403 (307–631) | 500 (295–666) | 357 (243–475) | 459 (318–587) | 468 (301–571) | 411 (341–499) |
|  | CID2 | 316 (257–466) | 420 (244–664) | 457 (350–509) | 465 (397–704) | 315 (272–567) | 584 (246–752) | 265 (143–397) | 244 (174–451) | 294 (240–376) | 400 (250–551) |
| TNFa (pg· mL^-1^) | CID1 | 0.9 (0.4–1.3) | 0.7 (0.4–1.1) | 0.9 (0.5–1.3) | 0.8 (0.5–0.8) | 0.8 (0.4–1.1) | 0.7 (0.4–1.6) | 0.9 (0.4–1.7) | 0.4 (0.2–1.5) | 1.0 (0.4–1.7) | 0.9 (0.4–1.9) |
|  | CID2 | 0.9 (0.4–1.2) | 0.6 (0.2–1.2) | 0.9 (0.7–1.0) | 0.6 (0.1–1.3) | 0.6 (0.2–1.0) | 0.6 (0.2–1.1) | 0.7 (0.5–1.4) | 0.6 (0.2–1.3) | 0.8 (0.4–1.6) | 0.9 (0.2–1.7) |
| GH (pg· mL^-1^) | CID1 | 86 (20–320) | 63 (15–208) | 56 (15–191) | 35 (10–82) | 74 (18–389) | 62 (11–640) | 149 (24–314) | 96 (43–702) | 147 (23–837) | 160 (12–322) |
|  | CID2 | 214 (71–596) | 91 (46–449) | 270 (56–743) | 67 (26–113) | 211 (46–416) | 296 (24–855) | 174 (93–510) | 123 (72–612) | 331 (92–1074) | 91 (21–386) |
| IGF1 (ng· mL^-1^) | CID1 | 6.9 (3.5–12.0) | 7.6 (4.2–13.8) | 6.1 (5.4–12.1) | 8.3 (1.3–12.8) | 8.4 (2.5–10.8) | 6.7 (1.8–14.3) | 5.9 (3.6–12.8) | 9.0 (5.5–14.8) | 7.1 (2.3–12.0) | 7.6 (4.7–13.0) |
|  | CID2 | 11.0 (5.0–15.8) | 12.0 (7.2–17.8) | 11.0 (5.3–16.0) | 10.7 (1.5–18.0) | 6.9 (1.5–15.3) | 9.6 (7.2–15.8) | 10.5 (7.3–15.8) | 13.0 (9.6–18.0) | 13.5 (6.3–16.5) | 12.0 (6.6–20.8) |
| IGFBP1 (ng· mL^-1^) | CID1 | 7.1 (3.5–13.9) | 4.9 (2.0–10.4) | 6.7 (2.8–8.9) | 1.9 (1.2–4.8) | 7.5 (4.1–22.1) | 8.0 (3.8–11.4) | 6.5 (2.5–13.9) | 6.4 (2.5–18.2) | 6.6 (3.2–15.9) | 4.7 (2.5–6.4) |
|  | CID2 | 6.6 (3.8–16.0) | 6.5 (3.4–13.8) | 8.0 (3.0–24.2) | 2.9 (1.6–6.0) | 7.2 (3.6–29.1) | 13.6 (8.8–17.2) | 7.2 (4.5–10.6) | 8.4 (4.9–18.0) | 6.2 (3.8–15.3) | 6.2 (3.6–10.5) |
| IGFBP3 (ng· mL^-1^) | CID1 | 671 (564–904) | 665 (525–943) | 735 (633–921) | 610 (362–915) | 651 (551–1131) | 701 (462–895) | 699 (501–843) | 579 (535–763) | 580 (527–865) | 996 (621–1303) |
|  | CID2 | 776 (573–1003) | 711 (572–906) | 807 (638–1112) | 649 (427–789) | 692 (382–865) | 745 (592–1038) | 825 (568–1052) | 708 (630–1059) | 795 (569–1003) | 703 (560–1188) |
| PEDF (µg· mL^-1^) | CID1 | 21.9 (14.7–42.1) | 19.5 (14.2–31.5) | 24.6 (14.9–55.9) | 19.5 (14.9–42.0) | 23.0 (14.9–60.2) | 19.5 (16.0–24.7) | 22.0 (12.9–27.5) | 14.5 (12.8–26.7) | 19.2 (14.6–29.4) | 28.0 (15.8–45.6) |
|  | CID2 | 16.9 (11.0–35.7) | 16.0 (10.7–26.8) | 20.4 (10.9–44.5) | 19.7 (9.5–34.7) | 15.6 (7.9–51.9) | 12.7 (12.3–34.8) | 12.8 (9.8–20.4) | 14.8 (9.5–17.7) | 17.3 (12.9–27.6) | 21.8 (9.3–53.5) |
| VEGFD (pg· mL^-1^) | CID1 | 447 (312–630) | 505 (343–605) | 320 (279–615) | 417 (311–523) | 447 (325–707) | 505 (325–642) | 391 (286–761) | 539 (365–724) | 526 (387–684) | 533 (365–596) |
|  | CID2 | 435 (386–700) | 548 (390–720) | 427 (326–435) | 574 (362–695) | 491 (373–675) | 553 (303–890) | 504 (390–799) | 556 (396–744) | 436 (396–903) | 511 (412–662) |
| GLP1 (pg· mL^-1^) | CID1 | 53.5 (40.3–67.8) | 51.0 (35.0–76.8) | 52.0 (38.8–65.5) | 49.5 (34.5–73.5) | 53.0 (39.0–82.0) | 55.5 (35.5–83.8) | 64.0 (41.5–68.0) | 44.5 (34.3–77.3) | 52.5 (40.3–58.8) | 52.0 (35.3–83.0) |
|  | CID2 | 48.0 (42.0–58.5) | 56.0 (40.5–72.3) | 46.0 (35.8–51.8) | 58.5 (45.5–63.8) | 52.5 (41.5–77.3) | 50.0 (35.5–76.8) | 50.0 (43.8–70.0) | 52.5 (45.0–74.5) | 46.0 (38.3–58.3) | 56.0 (40.0–79.0) |
| PP (pg· mL^-1^) | CID1 | 124 (96–180) | 126 (88–157) | 122 (100–146) | 134 (110–162) | 157 (82–216) | 95 (83–131) | 136 (90–182) | 147 (95–166) | 124 (92–145) | 117 (80–148)  118 (88–149) |
|  | CID2 | 108 (89–173) | 113 (89–148) | 100 (83–156) | 106 (79–143) | 107 (83–201) | 112 (86–145) | 105 (95–167) | 120 (93–185) | 112 (95–166) |  |

Values are expressed as median (interquartile range) from the fasted state.
